# Connective Tissue Growth Factor (CTGF/CCN2) Is Negatively Regulated during Neuron-Glioblastoma Interaction

**DOI:** 10.1371/journal.pone.0055605

**Published:** 2013-01-31

**Authors:** Luciana F. Romão, Fabio A. Mendes, Natalia M. Feitosa, Jane Cristina O. Faria, Juliana M. Coelho-Aguiar, Jorge Marcondes de Souza, Vivaldo Moura Neto, José Garcia Abreu

**Affiliations:** 1 Universidade Federal do Rio de Janeiro, Campus Macaé, Rio de Janeiro, Brazil; 2 Programa de Anatomia, Instituto de Ciências Biomédicas, Universidade Federal do Rio de Janeiro, Rio de Janeiro, Brazil; 3 Serviço de Neurocirurgia do Hospital Universitário Clementino Fraga Filho, Universidade Federal do Rio de Janeiro; Rio de Janeiro, Brazil; University of Florida, United States of America

## Abstract

Connective-tissue growth factor (CTGF/CCN2) is a matricellular-secreted protein involved in complex processes such as wound healing, angiogenesis, fibrosis and metastasis, in the regulation of cell proliferation, migration and extracellular matrix remodeling. Glioblastoma (GBM) is the major malignant primary brain tumor and its adaptation to the central nervous system microenvironment requires the production and remodeling of the extracellular matrix. Previously, we published an *in vitro* approach to test if neurons can influence the expression of the GBM extracellular matrix. We demonstrated that neurons remodeled glioma cell laminin. The present study shows that neurons are also able to modulate CTGF expression in GBM. CTGF immnoreactivity and mRNA levels in GBM cells are dramatically decreased when these cells are co-cultured with neonatal neurons. As proof of particular neuron effects, neonatal neurons co-cultured onto GBM cells also inhibit the reporter luciferase activity under control of the CTGF promoter, suggesting inhibition at the transcription level. This inhibition seems to be contact-mediated, since conditioned media from embryonic or neonatal neurons do not affect CTGF expression in GBM cells. Furthermore, the inhibition of CTGF expression in GBM/neuronal co-cultures seems to affect the two main signaling pathways related to CTGF. We observed inhibition of TGFβ luciferase reporter assay; however phopho-SMAD2 levels did not change in these co-cultures. In addition levels of phospho-p44/42 MAPK were decreased in co-cultured GBM cells. Finally, in transwell migration assay, CTGF siRNA transfected GBM cells or GBM cells co-cultured with neurons showed a decrease in the migration rate compared to controls. Previous data regarding laminin and these results demonstrating that CTGF is down-regulated in GBM cells co-cultured with neonatal neurons points out an interesting view in the understanding of the tumor and cerebral microenvironment interactions and could open up new strategies as well as suggest a new target in GBM control.

## Introduction

Neuron-glia interactions play fundamental roles during the development of the Central Nervous System (CNS). These interactions occur reciprocally from early to late stages of neurogenesis and gliogenesis, as well as during synapse establishment [1], [2]. Several lines of evidence illustrate the key participation of glial cells during neuronal network formation, in neurogenesis [3], [4], neuroblast proliferation [5], neuron migration [6], [7], neurite growth and guidance [8], [9], [10], [11], [12], as well as in myelination and synapse establishment [13], [14], [15], [16]. Neuronal cells can also control glial cell events, such as survival and proliferation by cell contact-mediated signaling, or by growth factor secretion, as shown in the interaction between axons and oligodendrocytes or Schwann cells (see [17] for review). *In vitro* studies have demonstrated that cell contact between astrocytes and neurons modulates astrocyte proliferation and differentiation through two distinct mechanisms [18], [19]. Neuronal membranes are sufficient to trigger inhibition of astrocyte proliferation, whereas astrocyte differentiation requires cell contact with living neurons [18], [19] and/or using TGFβ-1 as signaling [20], [21], [22]. Furthermore, we have also demonstrated that neurons induce glial astrocyte maturity by cell-cell contact and exchange of growth factors [23].

Despite this growing knowledge on normal neuronal-glial interactions, the effects of the interactions between normal neurons and tumors of glial origin, such as gliomas, are still an interesting subject for study. In this context, a study by Takano and co-workers focused on the effects of glutamate, secreted by C6 GBM, on neurons. Using a co-culture system, the authors demonstrated that neurons do not survive when in contact with C6 GBM cells secreting glutamate, and that this effect is abolished with a glutamate receptor antagonist [24].

GBM are the most common subtype of primary brain tumors in adults, and are characterized by their highly proliferative index, aggressiveness, invasiveness, and short patient survival, being considered the deadliest of human cancers [25], [26]. The control of glioma proliferation is a point of numerous studies, using, for example, different drugs [27], [28], [29], [30], [31], [32]. Glial cells, as well as GBM cells, can produce and modulate the synthesis of extracellular matrix (ECM) molecules in the brain [33], [34], [35], such as laminin, which may affect tumor aggressiveness and invasiveness. Indeed, our previous report shows that GBM express laminin and that neurons cultured onto these tumor cells remodeled the laminin architecture on the GBM surface [36].

More recently, much interest has been devoted to CTGF and cancer [37], [38], [39], [40]. CTGF belongs to a family of secreted ECM-associated proteins that are involved in the regulation of cellular functions, such as adhesion, migration, mitogenesis, differentiation and survival [41].

CTGF contains four different structural modules: an amino terminal insulin-like growth factor binding domain (IGFB), followed by the CR/vwc domain, a thrombospondin type 1 repeat (TSP-1), and a carboxyl terminal cystine knot (CT) domain [42], [43]. In the developing CNS of rodents, CTGF is expressed in the olfactory bulb, choroid plexus and dorsal root ganglia [44], [45]. In the adult CNS, CTGF is expressed in cortical astrocytes, cortical pyramidal cells, hippocampus, ependymal cells, tanicytes and in the white matter of spinal cord [46].

CTGF mRNA has also been detected in glioma and several human tumors cell lines derived from the nervous system and in tissue reorganization after brain injury, suggesting that it may play a role in the regulation of tumor invasiveness in the brain [46], [47], [48].

CTGF is involved in many biological events signaled by TGFβ, such as the control of collagen deposition and anchorage-independent growth induced by TGFβ in fibroblasts [49], [50]. The CTGF promoter contains a TGFβ responsive element [50], [51], and the CTGF molecule can positively modulate TGFβ-1 interaction with its cognate receptor and thus enhance signaling at low TGFβ concentrations [43].

CTGF gene expression and function can also be controlled by mitogen-activated protein (MAP) kinase pathways [52], [53]. The three major MAP kinase pathways, extracellular signal-regulated kinases (ERK), p38 and jun N-terminal kinase (JNK) serve as mediators and cross-talk links for a variety of signaling molecules and growth factors, and have been implicated in several cellular processes including cell growth, migration, proliferation, differentiation, survival and development. ERK is classically activated via the sequential activation of Ras G proteins, Raf kinases and MEK1 and 2. MEKs, in turn, phosphorylate and activate their only known substrates, ERK1 and 2. ERK1/2, also known as p44/42 MAPK, are proline-directed serine or threonine kinases which phosphorylate P-X-S/T-P sequences in a large number of intracellular substrates, leading to diverse cellular outcomes [54]. CTGF can activate MAPK signaling and its expression can be activated by ERK in different cell types, including fibroblasts, mesangial cells and smooth muscle cells [55], [56], [57], [58].

Despite CTGF expression in different CNS cell types and also in glioma-derived cells, the CTGF role in the interactions between normal and pathological CNS cells remains unknown.

Since we have demonstrated that neurons control glial differentiation [23], modulate the laminin architecture on the surface of human GBM [36], since CTGF is an important modulator of extracellular matrix proteins and stimulates tumor cell migration, the present study was undertaken to investigate whether neurons can modulate human GBM CTGF expression during neuron-human GBM cell line interaction in co-cultures. We found that GBM CTGF immunoreactivity and mRNA levels decrease dramatically when GBM95, GBM02 and U87 cells are cultured in contact with P0 neurons. To confirm that this was really and exclusively a neuronal effect only on the human GBM CTGF expression, the activity of the CTGF promoter was measured by a luciferase reporter (CTGF-lux), and it was demonstrated that the reporter was also down-regulated in GBM95/P0 and U87/P0 neurons co-cultures. TGFβ reporter assay showed that this signaling pathway is affected when GBM cells are in contact with neurons, however phosphor-Smad2 levels did not change. These results suggest that CTGF inhibition induced by co-culture with P0 neurons could be related to TGFβ but not to the canonical SMAD2/3 pathway. Interestingly, we also detected a decrease in phospho P44/P42-MAPK, indicating that MAPK signaling is also inhibited in the co-cultures. Moreover, in transwell assay, CTGF siRNA transfected GBM cells or GBM cells cultured with neurons showed a decrease in migration rate compared to controls. These data demonstrate that neonatal neurons are able to down-regulate CTGF expression in GBM95 cells, that this modulation seems not to depend on neuron-secreted soluble factors and that down-regulation may involve modulation of the TGFβ and MAPK signaling pathways and affects the migration/invasion of GBM cells.

## Methods

All animal experiments were approved by the Ethical Committee on the Use of Animals in Research of the Universidade Federal do Rio de Janeiro, under protocol DAHEICB015. All cell culture reagents and oligonucleotides were purchased from Invitrogen, and cDNA synthesis, PCR reagents and Luciferase assay kits were from Promega, unless specified otherwise. Anti-GFAP was purchased from Dako, anti-β tubulin III and anti-CNPase from Sigma, anti-CTGF from Torrey Pines, anti-P-SMAD2/3 and anti-P44/42MAPK from Cell Signaling. All solvents and reagents were of analytical grade.

### Primary cultures of rat neural cells

Effectively pure astrocyte cultures were obtained as described by Trentin et al. [59]. These cultures were used for GFAP expression analysis by RT-PCR and immunocytochemistry. Cultures enriched in rat neurons were obtained from embryonic 18-day-old and neonatal hemispheres, dissected as described above. Cells were plated in dishes or onto coverslips treated with poly-L-ornithine and maintained in serum-free DMEM. These cultures were kept at 37°C in a 5% CO_2_ atmosphere for no longer than 24 h. Both glial and neuronal cell cultures were assayed by immunocytochemistry with anti-GFAP and anti-β tubulin III antibodies to identify astrocytes and neurons, respectively.

### Human tumor cell culture

The Human GBM cell line (GBM95), GBM02 and GBM03 were obtained according to Faria et al. [36], following procedures established by the Brazilian Ministry of Health Ethic Committee (CONEP No. 2340). GBM95, GBM02 and GBM03 cell lines were derived from human samples and obtained with consent from the Brazilian Ministry of Health Ethic Committee and from written statements from the patients. Cells were plated in 35 mm or 16 mm well plates and the medium was changed every 3 days until confluence, when cells were usually split or frozen. The GBM U-87 and A-172, uterus U79 and lung GLC4 cell lines are described and were obtained in the cell line collection of ATCC.

### Co-cultures

GBM95 monolayers were used to grow neurons from E18 and P0 rat brains as described by Faria and collaborators [36]. Cells (5×10^4^) dissociated from E18 and P0 rat hemispheres as described above were plated on a GBM95 monolayer previously grown on 16 mm coverslips. For luciferase reporter assays, either 10^4^ or 2×10^5^ cells were plated onto the GBM95 monolayer. After 24 hours the medium was discarded and the co-cultures were fixed with 4% paraformaldehyde for immunocytochemistry or lysed with TRIZOL for RT-PCR.

### Conditioned medium from E18 and P0 neurons

Cells (3×10^5^) dissociated from E18 and P0 hemispheres were prepared and cultured as described above and gently washed three times with serum free medium. The cells were then incubated with the smallest possible volume of serum-free medium DMEM-F12 for 24 h. The conditioned medium enriched with cell-secreted molecules was collected, centrifuged at 1000 *x g* to discard cell debris and the supernatant was used immediately or stored at −80°C until use, as described previously [23].

### Immunocytochemistry

Immunostaining reaction was performed according to Faria and collaborators [36]. Briefly, fixed cultures or co-cultures were washed with PBS, permeabilized with 0.1% Triton X-100 in PBS for 5 min when necessary, and blocked for 1 hour with PBS containing 5% bovine serum albumin. Cultures were then incubated with rabbit anti-GFAP, Rabbit anti-CTGF or mouse anti-β tubulin III primary antibody for 1 hour at room temperature. Incubation with specific secondary antibodies conjugated either with fluorochromes Cy3 or FITC ensued for 1 hour at room temperature. After the PBS washes, slides were mounted and observed in a Nikon TE 2000 inverted microscope. Images were captured using a CoolSNAP-Pro (Media Cybernetics) digital camera.

### Western blot analysis of the cell lysates

GBM95, GBM02 or U87 monolayers or GBM95, GBM02 or U87 cells co-cultured with neurons maintained in 35 mm plates were lysed in 50 µl RIPA (0.05 M Tris-HCl pH 7.4, 0.15 M NaCl, 1% NP-40, 2 mM EDTA, 1 mg mL^−1^ pepstatin, 1 mM PMSF, 1 mM NaF, 1 mM Na_3_VO_4_) buffer. Protein concentrations was assayed by the Bradford method [60]. Before loading samples were mixed with 5X sample buffer containing 200 mM DTT 4% SDS, 125 mM Tris pH 6.8, 20% glycerol and 0.02% bromophenol blue. DNA was sheared in a 22-gauge needle syringe. Samples were heated during 5 minutes at 95°C and the proteins were separated on 9% SDS-PAGE gels. Proteins were then transferred to nitrocellulose membranes (Amersham Biosciences) in transfer buffer containing 25 mM Tris, 192 mM glycine, 0.1% SDS and 20% methanol. Membranes were blocked in 5% polyvinyl-pirrolidone (Sigma) for phospho-SMAD2/3 reaction or non-fat dry milk in 20 mM tris pH 7.6, 137 mM sodium chloride and 0.1% Tween 20 (Merk) for phospho-p44/42 MAPK reaction. Primary antibodies anti-Phospho-SMAD2/3 (Cell Signaling Technology), phospho-p44/42 MAPK (Cell Signaling Technology), total p44/42 MAPK (Cell Signaling Technology) or Anti-α tubulin diluted 1∶1000 in blocking solution were incubated with the membranes overnight at 4°C, followed by incubation with HRP-labeled secondary antibodies. The immunoblotting reaction was developed with Super Signal West Pico chemiluminescent substrate (Pierce) and exposed in Kodak X-OMAT film.

### Gene expression analysis by RT-PCR

GBM95, astrocyte, neuron cultures and GBM95/neuron co-cultures were lysed in TRIZOL and RNA extraction was performed according to the manufacturer's instructions. Before cDNA synthesis, RNA concentration was measured at 260 nm. For cDNA synthesis, 1 µg of RNA was reverse-transcribed using an ImProm II kit following the manufacturer's instructions for 1 h at 42°C. For semi-quantitative PCR, 3 µl of cDNA were mixed with PCR buffer containing 1.5 mM MgCl2, 0.2 mM dNTP, 0.2 µM oligonucleotide sense or anti-sense primers, 0.25 U Taq polymerase and nuclease-free water. The oligonucleotide sequences used were GAPDH (571 bp, 25 cycles) forward 5′ ATC ACC ATC TTC CAG GAG CG 3′ and reverse 5′ CCT GCT TCA CCA CCT TCT TG 3′, CTGF (236 bp, 30 cycles) forward 5′ GAA GGG CAA AAA GTG CAT CC 3′ and reverse 5′ GAC AGT TGT AAT GGC AGG CA 3′ [61], TGFβ-1 (161 bp, 30 cycles) forward 5′ GCC CTG GAC ACC AAC TAT TGC T 3′ and reverse 5′ AGG CTC CAA ATG TAG GGG CAG G 3′ [62]. cDNA fragments were separated by electrophoresis in a 2% agarose gel pre-stained with ethidium bromide (0.2 µg ml^−1^).

### Cell transfection and luciferase activity assay

GBM (1.5×10^4^) cells plated in a 96-well plate were transfected, immediately before attaching to the substrate, with CTGF-lux plasmids (luciferase reporter containing the entire CTGF promoter in pGL2) or 3TP-lux (luciferase reporter for TGFβ-1 pathway activity) and renilla luciferase for efficiency control, using the FuGENE 6 transfection reagent (Roche). The DNA/FuGENE mixture was maintained at room temperature for 20 min in DMEM-F12 (Invitrogen) without serum. This mixture was then added to the cells at the moment of plating with DMEM-F12 with 10% fetal bovine serum (Invitrogen). CTGF-lux/renilla GBM95, GBM02 or U87 transfected cells were co-cultured 24 h after the transfection with 1×10^4^, 5×10^4^ or 2×10^5^ freshly dissociated neurons from P0 rat cortex. 3TP-lux/renilla transfected cells were co-cultured 24 h after the transfection with 1×10^5^ freshly dissociated neurons from P0 rat cortex. After another 24-hour period, all cells (co-cultured or not) were lysed with passive lysis buffer (Promega) and luciferase activity was detected by adding the enzyme substrate provided in the Dual-Glo™ luciferase assay system (Promega), according to the manufacturer's instructions. The samples were assayed on a Veritas microplate luminometer (Turner BioSystems Inc., California, US). In order to normalize the data, the luciferase activity index was calculated by dividing firefly luciferase activity by renilla luciferase activity. Three independent experiments were conducted and the statistical analyses were performed using the GraphPad Prism 4.0 software.

### Transwell Cell migration assay

Cell migration was performed by transwell assay (BD Biosciences) according to the manufacturer's instructions. Control siRNA or CTGF siRNA GBM transfected cells were harvested 24 h after transfection. Then 5×10^4^ GBM transfected cells or GBM cells plus 5×10^4^ P0 neurons in serum free DMEM were seeded on a membrane (8.0-µm pore size) and inserted in a well of a 24-well plate. DMEM was added to the lower chamber of each well. After 24 h, cells in the upper chamber were removed by cotton swab and the cells that had reached the underside of the membrane were fixed with 4% paraformaldehyde and stained with hematoxylin-eosin (HE). The cells that located on the underside of the filter (10 fields/filter) were counted.

### Densitometry and Statistical Analysis

Densitometry of the immunocytochemistry images was performed in Image J (National Institute of Health). At least fifteen fields were counted per well. The experiments were done in triplicate, and each result shown here represents the mean of the three independent experiments. Statistical analysis was by t-test.

## Results

### CTGF expression in GBM

**Figure 1:**
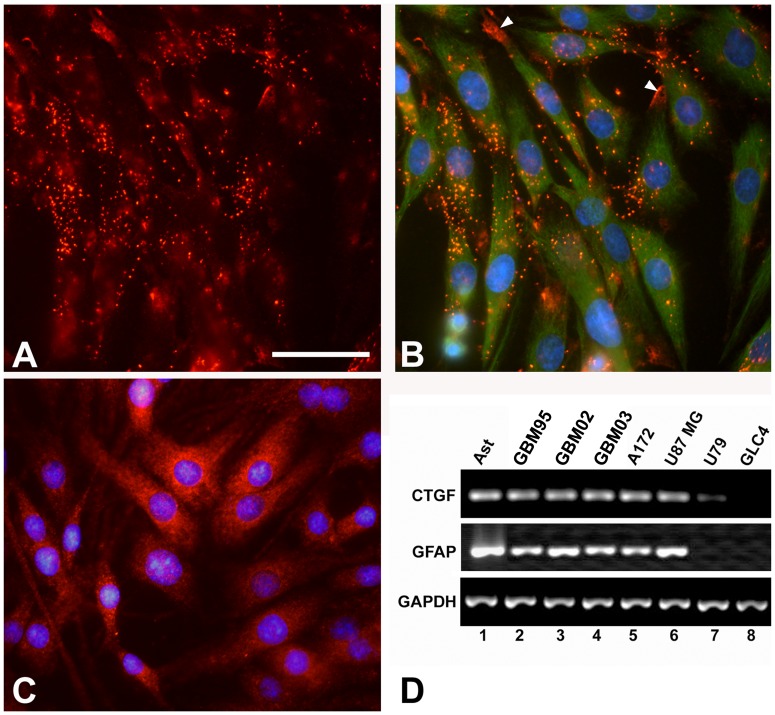
CTGF is expressed by GBM cells. Double immunostaining showing CTGF (A, B red) and vimentin (B green) expression. GFAP immunostaining of GBM95 cells (C). Nuclei-DAPI staining is shown in B and C. Scale bars (A, B, C) 50 µm. The arrowhead shows CTGF expression at the tip or borders of cell processes. RT-PCR analysis of CTGF and GFAP expression of astrocytes, GBM95, GBM02, GBM03, A172, U87 MG, U79 and GLC4 cells (lanes 1–6). GAPDH was used as loading control.

In order to investigate CTGF modulation in GBM cells we cultured GBM95, GBM02 and GBM03 cells previously generated in our laboratory [36] and first compared them to other tumor cell lines regarding CTGF expression. GBM95, GBM02 and GBM03 cultures had a similar aspect to cultures of known cell lines A172 or U87. All cultures showed fibrous morphology at conventional light microscopy (data not shown). After this comparison we then combined RT-PCR and immunocytochemistry in order to investigate CTGF expression in GBM95, GBM02, GBM03, A172 and U87 glioblastoma cell lines. Immunostaining of GBM95 with anti-CTGF showed punctate/aggregated patters of protein expression on the cell surface (Fig. 1A and B). In order to evidence cell morphology we double-stained GBM95 with antibodies against CTGF and the intermediate filament Vimentin. We observed an interesting and striking pattern of CTGF expression at the tip or borders of cell processes (Fig. 1A and B). The glial origin of GBM95 cells was confirmed by the GFAP expression mostly around the nuclei, distributed in the cytoplasm in a punctate pattern, different from the typical fiber-like organization found in rat astrocytes (Fig. 1C) [23], [63]. GFAP expression was also well detected by RT-PCR in GBM95, GBM02, GBM03, A172 and U87 cell lines, although an increased number of PCR cycles was required to obtain levels of expression similar to those detected in astrocytes (Fig. 1D, lanes 1–4). We found that astrocytes, as well as GBM95, GBM02, GBM03, A172 and U87 express similar levels of CTGF transcripts, but the non-glial-derived tumor cell lines U79 and GLC4 expressed lower levels or did not express CTGF (Fig. 1D). These data show that GBM95 expresses the glial marker GFAP as well as CTGF, and is, therefore, a suitable model for the study of CTGF modulation in neuron-glioblastoma interaction.

### CTGF expression is down-regulated in GBM cells co-cultured with rat neurons

**Figure 2:**
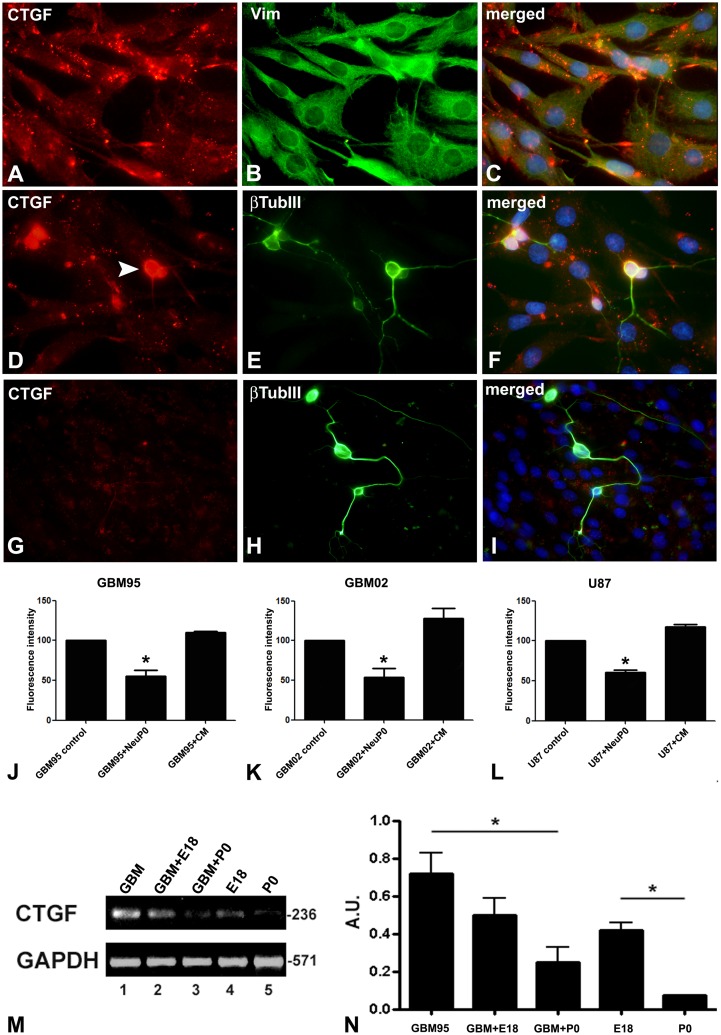
CTGF immunoreactivity is decreased and CTGF mRNA is down-regulated in co-cultures of GBM cells with neurons. Immunocytochemistry showing CTGF expression (red) in GBM 95 cultures (A, C) and co-cultures of GBM95 with E18 (D, F) or P0 neurons (G, I). Stained neuron β tubulin III (green) are shown in (E, H). The arrowhead shows a CTGF stained E18 neuron (D). Notice that CTGF staining decreased in co-cultures (comparing A with D and G). Bar 50 µm. Densitometry of the immunocytochemistry images show a decrease in the intensity of CTGF staining of GBM95, GBM02 or U87 co-cultured with P0 neurons (J–L) (p<0.05). RT-PCR analysis of CTGF expression in GBM95 co-cultured with E18 or P0 neurons (M). Base pairs numbers appears on the right side of each gel. GAPDH was used as loading control. Histogram expressing arbitrary units (A.U) products obtained from CTGF over GAPDH of the PCR bands (N). CTGF expression of GBM95 co-cultured with P0 neurons was decreased when compared to pure GBM95 cultures.

In order to investigate if normal embryonic or neonatal rat neurons could modulate the expression of CTGF in GBM cells, as occurred with laminin on this GBM [36], we co-cultured E18 and P0 onto GBM95, GBM02 or U87 cell carpets and performed double immunocytochemistry with anti-CTGF antibody and anti-β tubulin III (Fig. 2).

We observed a decrease in CTGF staining of GBM95, GBM02 and U87 cells when these were co-cultured with embryonic neurons and a more dramatic decrease when these were co-cultured with neonatal neurons (Fig. 2A, D and G). The intensity of the fluorescence was measured and we could observe approximately 50% reduction when neurons were co-cultured with GBM95, GBM02 and U87 glioblastoma cell lines (Fig. 2J–L). We did not observe any decrease in the intensity of the fluorescence when GBM95, GBM02 and U87 glioblastoma cell lines were cultured in presence of conditioned medium from P0 neurons (Fig. 2J–L). Immunocytochemistry and RT-PCR were also performed for GBM95 cell line culture in presence of conditioned medium from E18 and P0 neurons and no differences were observed (Figure S1). Surprisingly, we observed a strong CTGF staining in embryonic neurons, mostly in the cell body (Fig. 2D and F). Such staining was also observed when embryonic neurons were cultured in a tumor-free condition (data not shown). However, CTGF staining in P0 neurons was very weak, suggesting that these neurons had lost CTGF expression (Fig. 2G and I).

RT-PCR analysis showed that the level of CTGF mRNA confirmed that CTGF expression was diminishing in the P0 neurons and GBM95 cell co-cultures in comparison to pure GBM95 cell cultures (Fig. 2M, lanes 1 and 3 and N), and decreased, albeit slightly, when E18 neurons were co-cultured onto GBM95 cells (Fig. 2M, lanes 1 and 2 and N). We did not observe CTGF mRNA expression in P0 neurons (Fig. 2M, lane 5 and N), but this expression was detectable in E18 neurons (Fig. 2M, lane 4 and N). In order to address the total RNA dilution between cultured and co-cultured samples, we counted the number of rat neurons on the GBM95 cells and found that they represent 5% to 8% of the total cell population on the plate (data not shown). We also stained these rat brain cells co-cultured with GBM95 and found that they consisted of 95% β tubulin III-positive and 5% GFAP-positive cells (data not shown). In order to investigate if the CTGF modulation promoted by P0 neurons was a specific feature of GBM95 cells, we performed co-culture experiments using normal astroglial monolayers as substrate for E18 and P0 Neurons. As seen in GBM cells, CTGF expression was decreased in astrocytes co-cultured with both E18 and P0 neurons (Figure S2). These results show that CTGF protein and mRNA expression is inhibited in GBM cells co-cultured with neonatal neurons.

### Neuron-GBM interaction inhibits CTGF promoter activation

**Figure 3:**
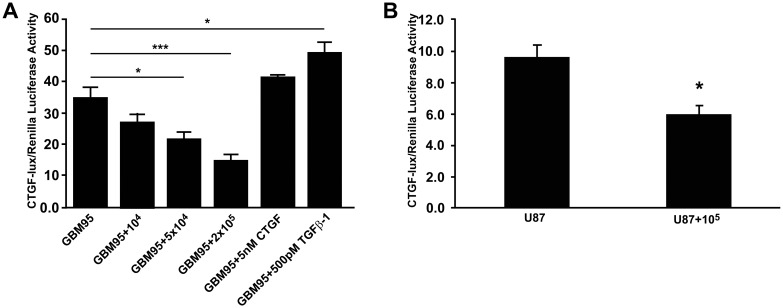
CTGF promoter activity is inhibited when neurons are co-cultured with GBM cells. Graph shows Firefly/Renilla luciferase activity of GBM95 or U87 cells transfected with CTGF-lux and Renilla plasmids co-cultured with different amounts of freshly dissociated neurons. Note that 5×10^4^ neurons were able to inhibit more than 40% of luciferase activity (comparing first to third bar, p<0.05) and 2×10^5^ neurons were able to inhibit more than 50% of luciferase activity controlled by the CTGF promoter (A) (comparing first to fourth bar, p<0.01). As a positive control 0.5 nM of TGFβ-1 was used and we could observed an increase of almost 40% of luciferase activity (comparing first to fifth bar, p<0.05). 1×10^5^ P0 neurons were able to inhibit almost 40% of luciferase activity when co-cultured with U87 cells (B) (p<0.05).

Since our results suggested that neuron-GBM interaction negatively regulates CTGF transcription in GBM cells, we performed a luciferase reporter assay to test if neurons could directly inhibit the activation of the CTGF promoter (Fig. 3), without a dilution effect of the co-culture. First, we transfected GBM95 and U87 cells with a luciferase reporter controlled by the full length CTGF promoter (CTGF-lux). Transfected GBM95 and U87 cells were co-cultured with 1×10^4^, 5×10^4^, 1×10^5^ or 2×10^5^ freshly dissociated neurons from P0 rat cortex and luciferase activity was measured after 24 h. GBM95 co-cultures with 1×10^4^ neurons showed 20% inhibition of the luciferase activity, co-cultures with 5×10^4^ neurons inhibited approximately 40% luciferase activity and co-cultures with 2×10^5^ neurons showed a more striking result with 60% inhibition (Fig. 3A). In addition, as a positive control for the luciferase reporter assay, we incubated GBM95-transfected cells with 5 nM CTGF or 0.5 nM TGFβ-1 recombinant proteins (Fig. 3A). We also performed this reporter assay with U87, a commercial GBM cell line. 1×10^5^ neurons were able to inhibit approximately 30% luciferase activity (Fig. 3B).These results demonstrate that neuron-GBM interaction negatively regulates transcription via CTGF promoter.

### Inhibition of CTGF in GBM cells by P0 neurons does not require TGFβ/SMAD2/3 signaling

**Figure 4:**
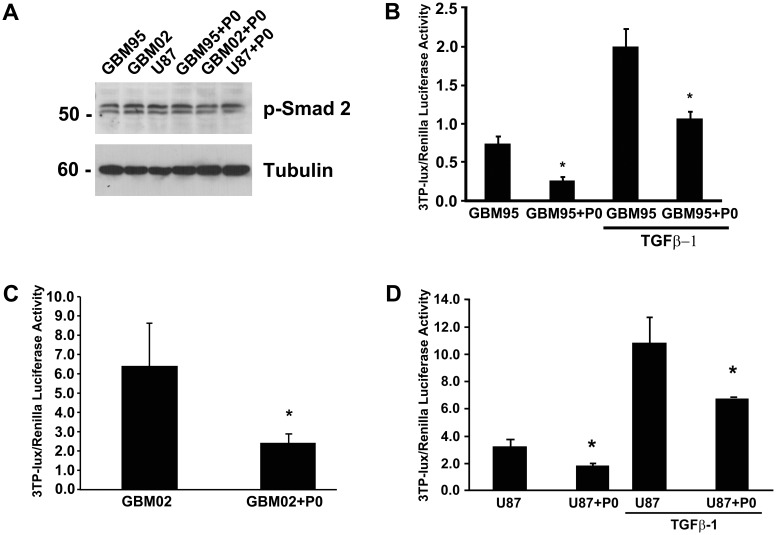
Inhibition of CTGF in GBM cells by P0 neurons do not require TGFβ/SMAD2/3 signaling. (A) Western blot analysis of the cell extracts from GBM95, GBM02 or U87 and GBM95, GBM02 or U87 co-cultured with P0 neurons. From top to bottom, the pictures show membranes reacted with anti-phospho SMAD2/3 and anti-α tubulin antibodies. Molecular weight in kDa is shown on the left side of the picture. (B–D) Graphs show Firefly/Renilla luciferase activity of GBM95 (B), GBM02 (C) or U87 (D) cells transfected with 3TP-lux and Renilla plasmids were cultured alone or P0 neurons, in presence or absence of TGFβ-1 (1 nM). Each point represents the average of three independent experiments done in triplicate.*p<0.05 (in comparison to the control).

We tested whether the TGFβ/SMAD2/3 signaling pathway was involved in CTGF inhibition by P0 neurons by analyzing Smad2/3 phosphorylation in western blots of GBM95, GBM02 or U87 and GBM95/P0, GBM02/P0 or U87/P0 co-cultures (Fig. 4A). In this assay, neurons were maintained in co-cultures with GBM95 cells for 30 min in order to detect SMAD 2/3 phosphorilation. However, no differences were observed in SMAD2/3 phosphorylation between GBMs alone and GBMs co-cultured with P0 neurons (Fig. 4A). In order to detect TGFβ signaling with a more sensitive assay GBM95 cells were transfected with 3TP-lux luciferase reporter plasmid. 1×10^5^ neurons were able to inhibit 60% and 40% luciferase activity without or with 1 nM exogenous TGFβ-1 protein (Fig. 4B). The same results were found to be true for other GBM cell lines such as GBM02 (established by our group and described in [36]) and U87 (Fig. 4C and D). In GBM02 the decrease was approximately 60% without TGFβ-1 (Fig. 4C). Exogenous TGFβ-1 did not activate 3TP-lux reporter in GBM02 suggesting that TGFβ signaling is already high in this cell line (Data not shown). In U87 the decrease was approximately 40% with or without exogenous TGFβ-1 protein (Fig. 4D). These results suggest that CTGF inhibition induced by co-culture with P0 neurons could be related to TGFβ but not to the canonical SMAD2/3 pathway

### p44/42-MAPK phosphorylation decreases during GBM95/P0 interaction

**Figure 5:**
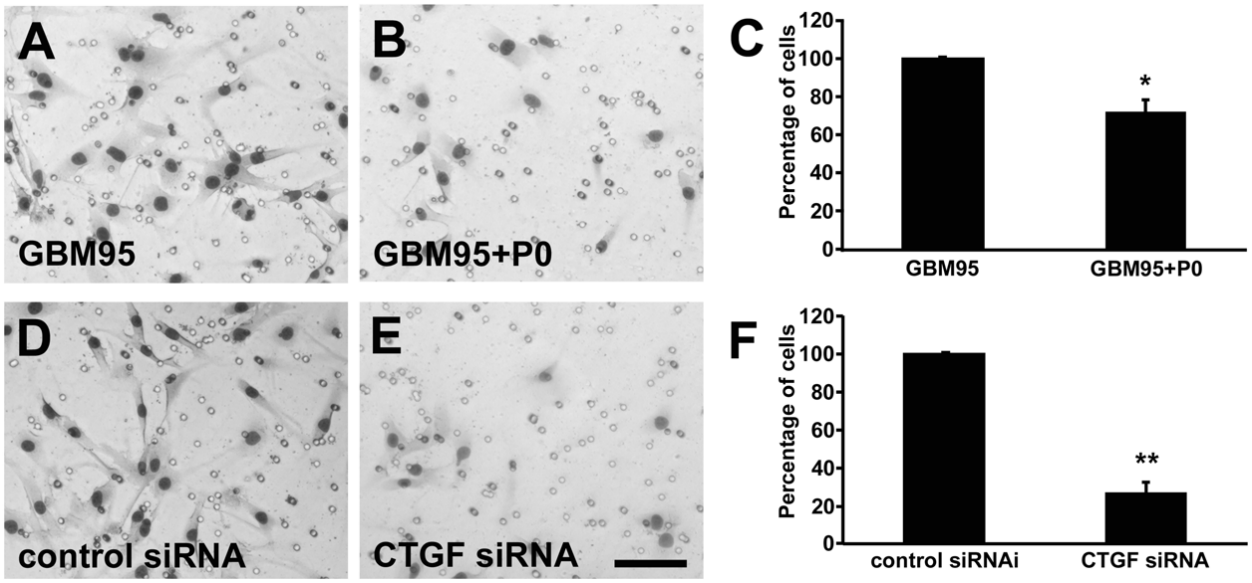
Downregulation of CTGF inhibits GBM95 cell migration. Representative images of HE stained migrated cells. GBM95 cells (A) GBM95 platted with P0 neurons (B) Graph showing the percentage of cells that migrate in each condition (C). GBM95 transfected with control siRNA (D) or siRNA to downregulate CTGF expression (E). Statistical analysis of the effects of downregulation of CTGF on the GBM95 migration (F). Transwell migration of co-cultures of GBM95 with P0 neurons and GBM95 siRNA to downregulate CTGF shows a significant reduction in motility when CTGF levels are reduced. Results are mean±SD from three experiments each performed in duplicate. The numbers of invasive cells were significant different from the control group (*p<0.05 and **p<0.01).

Since the MAPK signaling pathway has been also shown to modulate CTGF expression, and seems to be related to some of TGFβ actions, we then measured the levels of phospho p44/42-MAPK by western blot (Figure S3). We detected p44/42-MAPK and its phosphorylated form in both GBM95 and GBM95/P0 co-cultures, but not in pure P0 neuron cultures (Figure S3A, lanes 1–3 and B). However, phospho p44/42-MAPK levels were decreased in GBM95 co-cultured with P0 neurons for 30 min when compared to GBM95 cells alone (Fig. 5C, lane 2 and D). This result indicates that the inhibition of CTGF expression in GBM95 cells by P0 neurons might be mediated by a negative regulation of MAPK/ERK signaling.

### Neuron-GBM interaction inhibits GBM cells migration

**Figure 6:**
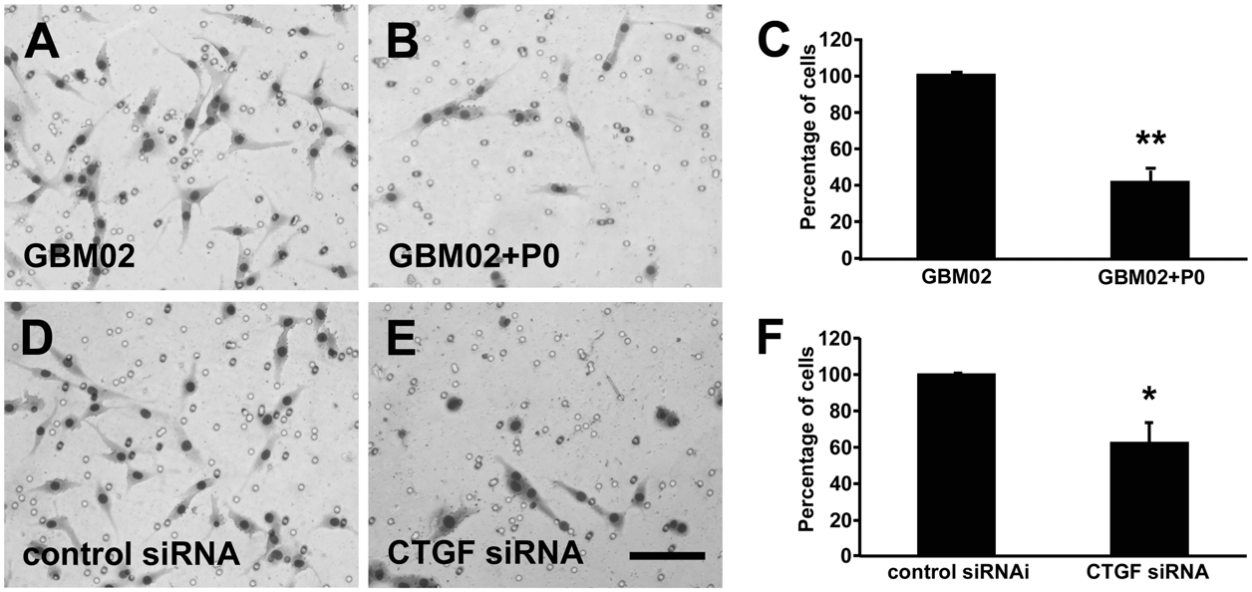
Downregulation of CTGF inhibits GBM02 cell migration. Representative images of HE stained migrated cells. GBM 02 cells (A) GBM02 platted with P0 neurons (B) Graph showing the percentage of cells that migrate in each condition (C). GBM02 were transfected with control siRNA (D) or siRNA to down regulate CTGF expression (E). Statistical analysis of the effects with P0 neurons and downregulation of CTGF on the GBM02 migration (F). Downregulation of CTGF dramatically reduced GBM02 cell migration in vitro. Results are mean±SD from three experiments each performed in duplicate. The numbers of invasive cells were significant different from the control group (*p<0.05 and **p<0.01).

**Figure 7:**
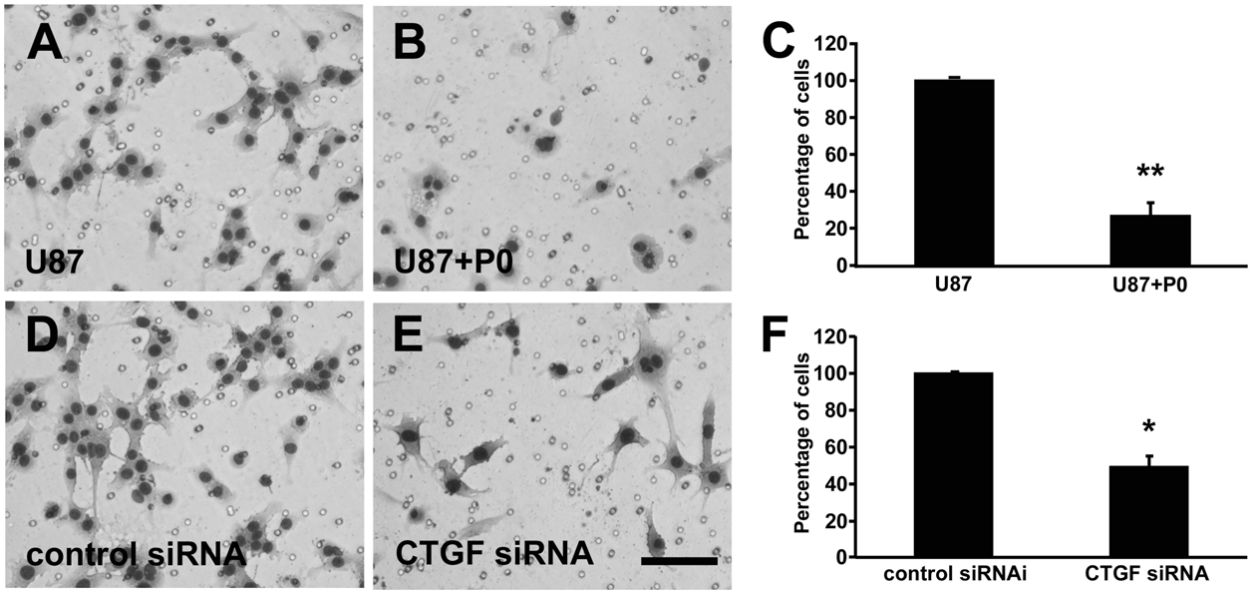
Downregulation of CTGF inhibits U87 cell migration. Representative images of HE stained migrated cells. U87 cells (A) U87 platted with P0 neurons (B) Graph showing the percentage of cells that migrate in each condition (C). U87 were transfected with control siRNA (D) or siRNA to down regulate CTGF expression (E). Statistical analysis of the effects with P0 neurons and downregulation of CTGF on the GBM02 migration (F). Downregulation of CTGF dramatically reduced U87 cell migration in vitro. Results are mean±SD from three experiments each performed in duplicate. *p<0.05 and **p<0.01, statistically significant difference.

Since CTGF is implicated with migration and invasion of several types of tumor, including GBM, we tested in a transwell migration assay if CTGF down regulation induced by neuron-GBM interaction could impair migration of GBM95 cells. 5×10^4^ cells were platted in the upper compartment of a transwell, alone or with 5×10^4^ P0 neurons. After 24 h, the cells were fixed and the number of cells on the other side of the membrane was counted. We observed that the migration rate of GBM95 cells platted with P0 neurons decreased approximately 25% compared to GBM95 cells platted alone (Fig. 5 A–C). The same experiment was performed with GBM95 cells transfected with CTGF siRNA or with control siRNA. We observed that the migration rate of CTGF siRNA transfected GBM95 cells decreased approximately 70% compared to cells transfected with control siRNA (Fig. 5 D–F). We also challenged GBM02 and U87 migration in the presence of P0 neurons. The migrations rate of these cell lines decreased approximately 60% and 70% respectively compared to the migration of the cells alone (Fig. 6 A–C and Fig. 7 A–C). CTGF siRNA transfected GBM02 and U87 also showed 40% and 50% lower migration rates respectively compared to control siRNA transfected cells (Fig. 6 D–F and Fig. 7 D–F). These results point out an important relation between GBM cells and healthy neurons implicating in reduced migration rate of the tumor cells and suggest that the decrease in migration rate might be related to the down regulation of CTGF expression induced by neurons.

## Discussion

In the present study we investigated the modulation of the expression of the matricellular molecule CTGF during neuron-GBM interaction *in vitro*. Our results show that CTGF transcription and transduction is negatively regulated in GBM cells in the presence of normal neonatal neurons. CTGF down-regulation seems to require GBM/neuron contact and do not seems to involve the TGFβ/SMAD2/3 pathway, a major regulator of CTGF expression. It might involve at least one effector of MAPK signaling, p44/42 MAPK (ERK1/2), which is negatively affected during this interaction.

In our previous studies, we demonstrated that human GBM cells (GBM95, GBM02 and GBM03) can recapitulate neuron-normal astrocyte interaction by supporting neurite growth. We also showed that laminin has its distribution remodeled from a fiber-like to an aggregate pattern in GBM95 cells or normal astrocytes co-cultured with neonatal neurons [36], suggesting that this is a persistent property of the neurons. Here we further describe the neuron-GBM interaction. Firstly, we show that GBM cells synthesize the secreted ECM-associated protein CTGF and its RNA. In this sense, it has been demonstrated *in vivo* that not only neoplasic GFAP-positive astrocyte-like cells express CTGF, but that even GFAP-negative cells in the tumor can also express CTGF [37]. Despite the differences between *in vivo* and *in vitro* microenvironments, it seems that GBM cells maintain their ability to express glial phenotypes and still present, as a “biological memory”, some functional properties of non-neoplasic glia.

Further, CTGF contains a unique TGFβ-inducible element on its promoter, pointing to TGFβ-1 as a stronger inducer of CTGF [64]. In addition, we demonstrated that the same protein domain of CTGF binds TGFβ-1 and BMP2/4, but that these interactions result in different modulations of these signaling pathways [43], [65]. CTGF enhances the binding of TGFβ-1 to its cognate receptors and increases SMAD2/3 phosphorylation [43]. In this context, GBM95 cells are responsive to TGFβ-1 leading to the expression of high levels of CTGF transcripts (Data not shown). Ernst and co-workers used a spheroidal culture, enriched with GBM tumor-initiating cells, to show that, under differentiating conditions, CTGF is up-regulated [66]. Their results suggest that CTGF and possibly the TGFβ signaling pathway could be involved with GBM tumorigenesis. In this context, we demonstrated that CTGF transcription in GBM cultures was decreased in the presence of P0 neurons and that TGFβ signaling was also inhibited during neuron-glioblastoma interactions in GBM95, GBM02 and U87 transfected with 3TP-lux reporter. On the other hand, SMAD2/3 phosphorilation levels did not change when GBM cell lines were co-cultured with P0 neurons. These results suggest a possible involvement of other TGFβ pathway. Yue and collaborators showed that Ras/ERK pathway is necessary for the SMAD1 activation of 3TP-lux reporter [67]. We cannot rule out the importance of other signaling pathways specially the contact-mediated ones, since increasing numbers of neurons cultured over GBM cells inhibited CTGF promoter, but neuronal conditioned medium had no effect.

MAPK signaling pathways are known to be involved with CTGF actions such as proliferation and differentiation and in CTGF-mediated adhesion [55], [58], [68], [69]. These studies have focused mainly on cytoskeletal rearrangement/integrity and focal adhesion induced by CTGF, triggered by its interaction with ECM-related molecules [56], [70]. It is believed that TGFβ is able to signal via Ras and Rac proteins and activate MAP kinases, including ERK1 and 2 and JNK [52]. Chen and coworkers showed that maximal TGFβ induction of CTGF expression requires synergy between SMAD and Ras/MEK/ERK signaling [53]. Shimo and coworkers [71] showed that CTGF is important for chondrocyte maturation and that its expression during this process was regulated positively by ERK1/2 and negatively by p38 MAP kinases. We demonstrated that contact with P0 neurons down-regulates CTGF expression in GBM95 cells concomitantly with a decrease in ERK1/2 phosphorylation, suggesting a possible influence of different signaling pathways during neuron-glioblastoma interactions.

It is striking that the strongest CTGF inhibition observed in GBM cells was induced by neonatal but not by embryonic neurons. One possible explanation is that contact-mediated interactions with embryonic versus neonatal neurons is due to development illustrating new windows of interactions and naturally involving different molecules. This development compromise was demonstrated by our previous studies, that pointed out that astrocytes are a better substrate for embryonic rather than post-natal neurons and in this same development manner we could also illustrate that neurons can secrete specific soluble factors which induce GFAP promoter gene, suggesting that neuron-glia interaction can induce the astrocytic differentiation program [23]. Although GBM cells support neurite outgrowth of both embryonic and post-natal neurons as normal astrocytes, it seems that the GBM cell line does not interpret all the signals produced by embryonic or post-natal neurons as astrocytes do [23], [36].

Glioma cell invasion is a complex and multistep mechanism involving a large array of molecules and cell-cell and cell-ECM interactions. Glioblastoma is usually located in one of the cerebral hemispheres, with an epicenter in the white matter, but frequently bilaterally invades the subcortical white matter through the corpus callosum. A rapid spread is also observed into the white matter of the internal capsule, fornix and anterior commissure [25]. Thus, the modulation of signaling pathways in tumor-normal brain interactions is likely to be of great importance in explaining specific glioma invasion patterns, including along white matter tracts, into the subpial space and along the external walls of blood vessels.

It has been shown that breast cancer cell subpopulation with highly metastatic activity over-express the CTGF gene and other secreted and cell surface molecules that act cooperatively in a multigenic osteolytic metastasis [72]. Although very little is known regarding CTGF function on GBM cells it has been found that CTGF mRNA is 58% increased in primary gliomas compared to normal brain samples [38]. CTGF was associated with GBM migration *in vitro* [73], [74], and, more recently, with infiltration *in vivo*, through activation of the Tyrosine Kinase Receptor Type A (TrkA) and down-regulation of E-cadherin [75]. Furthermore, there was a significant correlation between CTGF mRNA levels in these tumors with tumor grade and pathology, suggesting that CTGF may play a role in glioma progression [38], [39]. Thus, our results pointing CTGF inhibition in GBM through neuronal contact highlight the importance of the interactions between tumor and normal cells in the progression and invasiveness properties of GBM. Then, this opens a new discussion implicating neurons on the GBM progression. Glioblastoma occurs most often in the subcortical white matter of the cerebral hemispheres. Since tumor infiltration often extends into the cortex [25], it is possible to argue if, in the first installation step, neurons could control CTGF expression of the small tumor and as consequence control tumor migration and/or invasion. Tumoral progression is slow in the initial phase of a small tumoral mass. Later, as the tumor grows, it could secrete enough toxic glutamate and kills the neuronal cells [24], [76], [77]. Then, CTGF neuronal inhibition might be abolished. So, later, a fatal and very large tumor, that bypasses the neuronal control, could promote neuronal death and occupies a larger area of the brain.

In summary, our present results show that neuron-GBM interaction negatively modulates CTGF. This inhibition occurs at the transcriptional level, seems to depend on cell contact and seems not to require TGFβ/SMAD2/3. Colllectively, this data could contribute to a better knowledge of GBM behavior in the brain microenvironment, and strongly suggest that glial tumors can maintain fundamental properties of glial cell interaction with neurons.

## Supporting Information

Figure S1 **Conditioned medium from E18 and P0 neurons does not affect CTGF expression in GBM.** CTGF immunocytochemistry of GBM95 cells (A) treated with conditioned medium (cm) from E18 (B) or P0 neurons (C). Bar 50 µm. (D) RT-PCR Analysis of CTGF expression in GBM95 cultured for 24 h with cmE18 and cmP0 neurons. Base pairs number appears on the right side of each gel. GAPDH was used as loading control. CTGF expression was not changed in GBM95 cells cultured in neuronal conditioned medium.
(TIF)

Figure S2 **CTGF mRNA is down-regulated in co-cultures of astrocytes cells with neurons.** RT-PCR Analysis of CTGF and TGFβ-1 expression in embryonic astrocytes (lane 1) or astrocytes and neurons co-cultures (lanes 2 and 3). Base pairs number appears on the right side of each gel. GAPDH was used as loading control. CTGF expression of embryonic astrocytes co-cultured with E18 and P0 neurons was also decreased when compared to pure embryonic astrocytes cultures.
(TIF)

Figure S3 **Phospho-p44/42 MAPK decreases in GBM95 cells co-cultured with P0 neurons.** (A) Western blot analysis of the cell extracts from GBM95 (lane 1) and GBM95 co-cultured with P0 neurons (lane 2) and only P0 neurons (lane 3). From top to bottom, membranes were reacted with anti-phospho-p44/42 MAPK and anti-p44/42 MAPK. Histogram expressing the AU products from p44/42 MAPK over MAPK bands (B). Levels of total p44/42 MAPK did not change, but phospho p44/42 decreased in GBM95/P0 co-cultures. Molecular weight in kDa is shown on the left side of the picture. Each experiment is representative of at least three independent experiments. *p<0.05 compared to control.
(TIF)
