# Supplementary figures and images for: Connective Tissue Growth Factor (CTGF/CCN2) Is Negatively Regulated during Neuron-Glioblastoma Interaction
Source: PLoS One. 2013 Jan 31;8(1):e55605. doi: 10.1371/journal.pone.0055605 (PMC3561339; doi:10.1371/journal.pone.0055605)

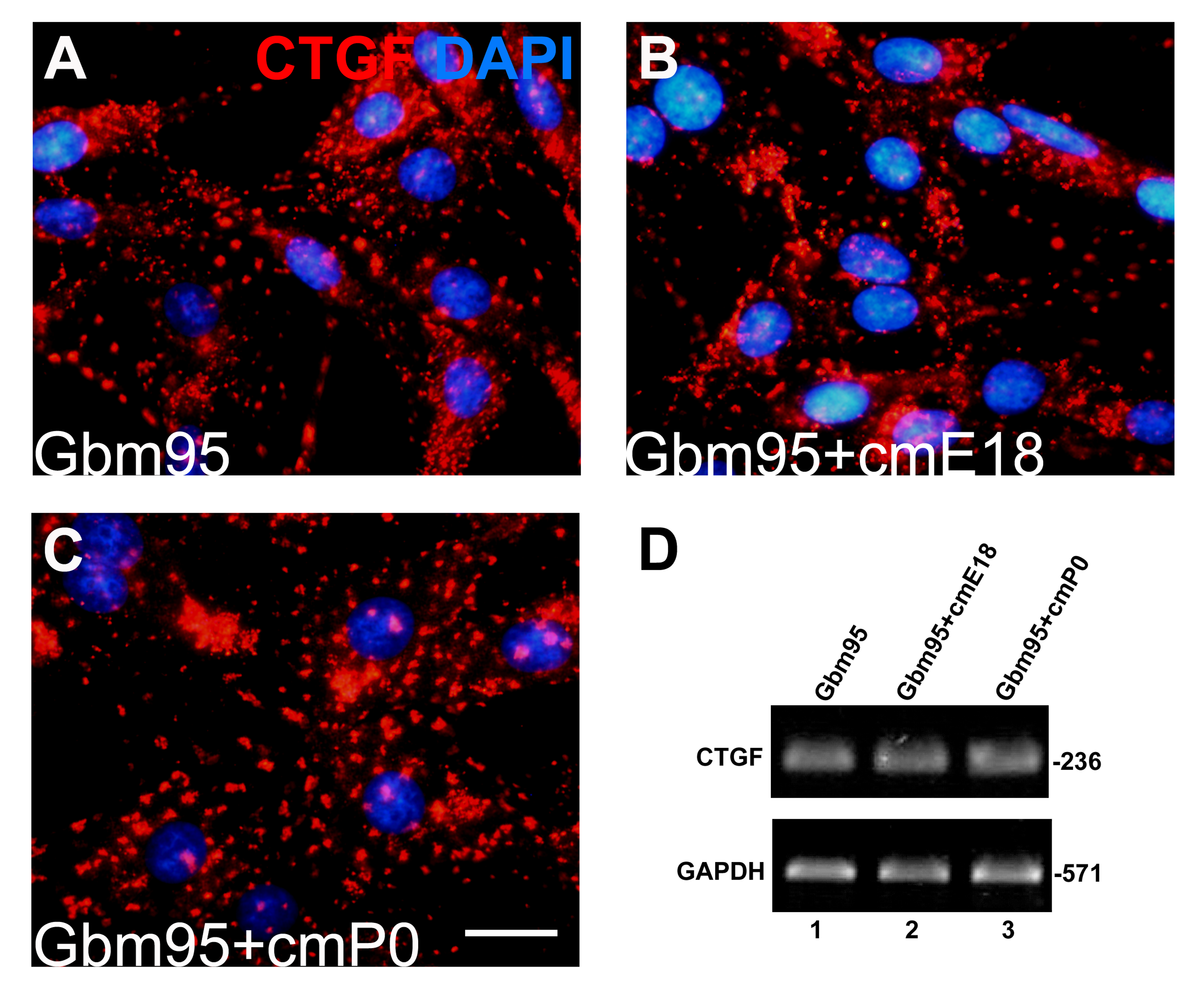

Supplement: Figure S1 — Conditioned medium from E18 and P0 neurons does not affect CTGF expression in GBM. CTGF immunocytochemistry of GBM95 cells (A) treated with conditioned medium (cm) from E18 (B) or P0 neurons (C). Bar 50 µm. (D) RT-PCR Analysis of CTGF expression in GBM95 cultured for 24 h with cmE18 and cmP0 neurons. Base pairs number appears on the right side of each gel. GAPDH was used as loading control. CTGF expression was not changed in GBM95 cells cultured in neuronal conditioned medium. (TIF) [file pone.0055605.s001.tif]

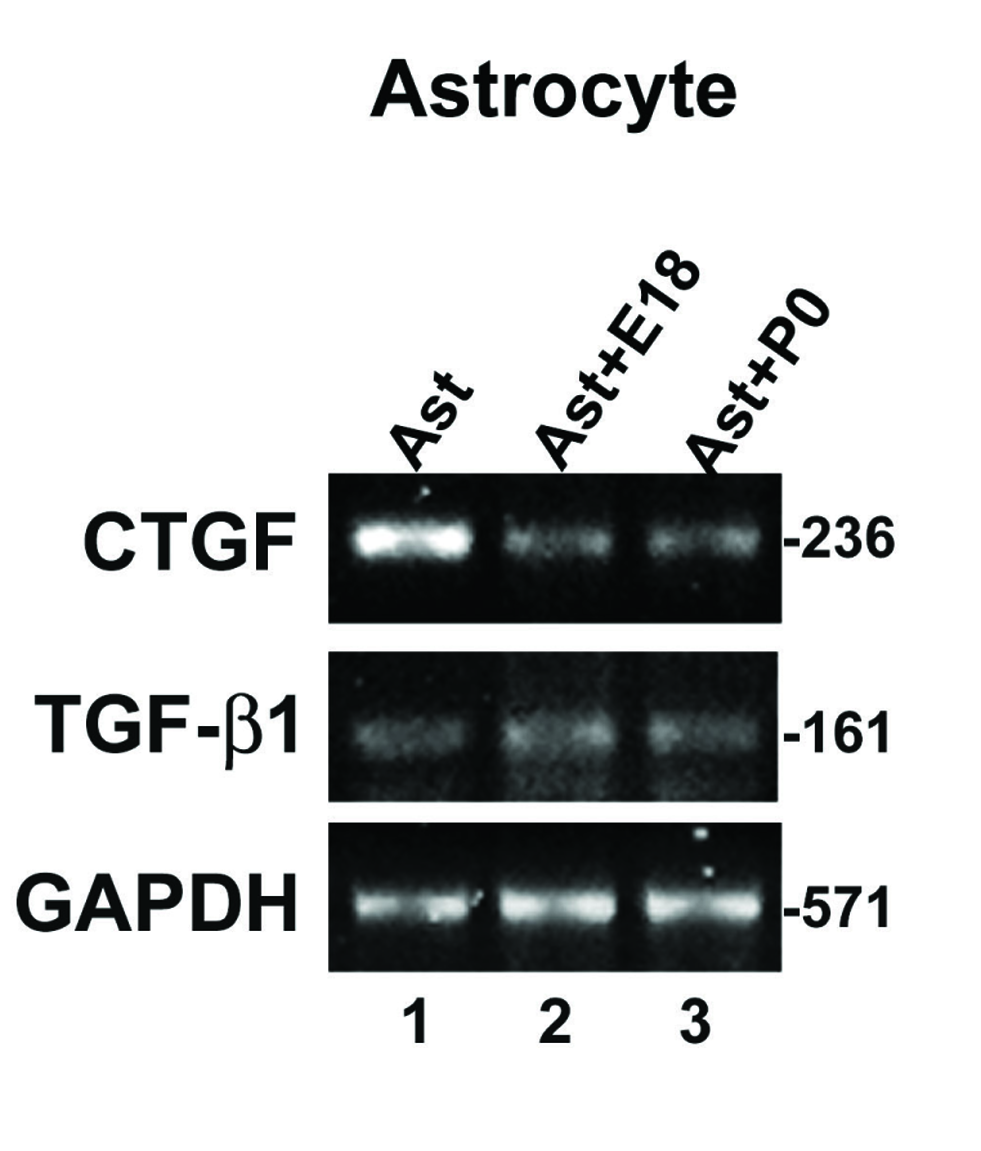

Supplement: Figure S2 — CTGF mRNA is down-regulated in co-cultures of astrocytes cells with neurons. RT-PCR Analysis of CTGF and TGFβ-1 expression in embryonic astrocytes (lane 1) or astrocytes and neurons co-cultures (lanes 2 and 3). Base pairs number appears on the right side of each gel. GAPDH was used as loading control. CTGF expression of embryonic astrocytes co-cultured with E18 and P0 neurons was also decreased when compared to pure embryonic astrocytes cultures. (TIF) [file pone.0055605.s002.tif]

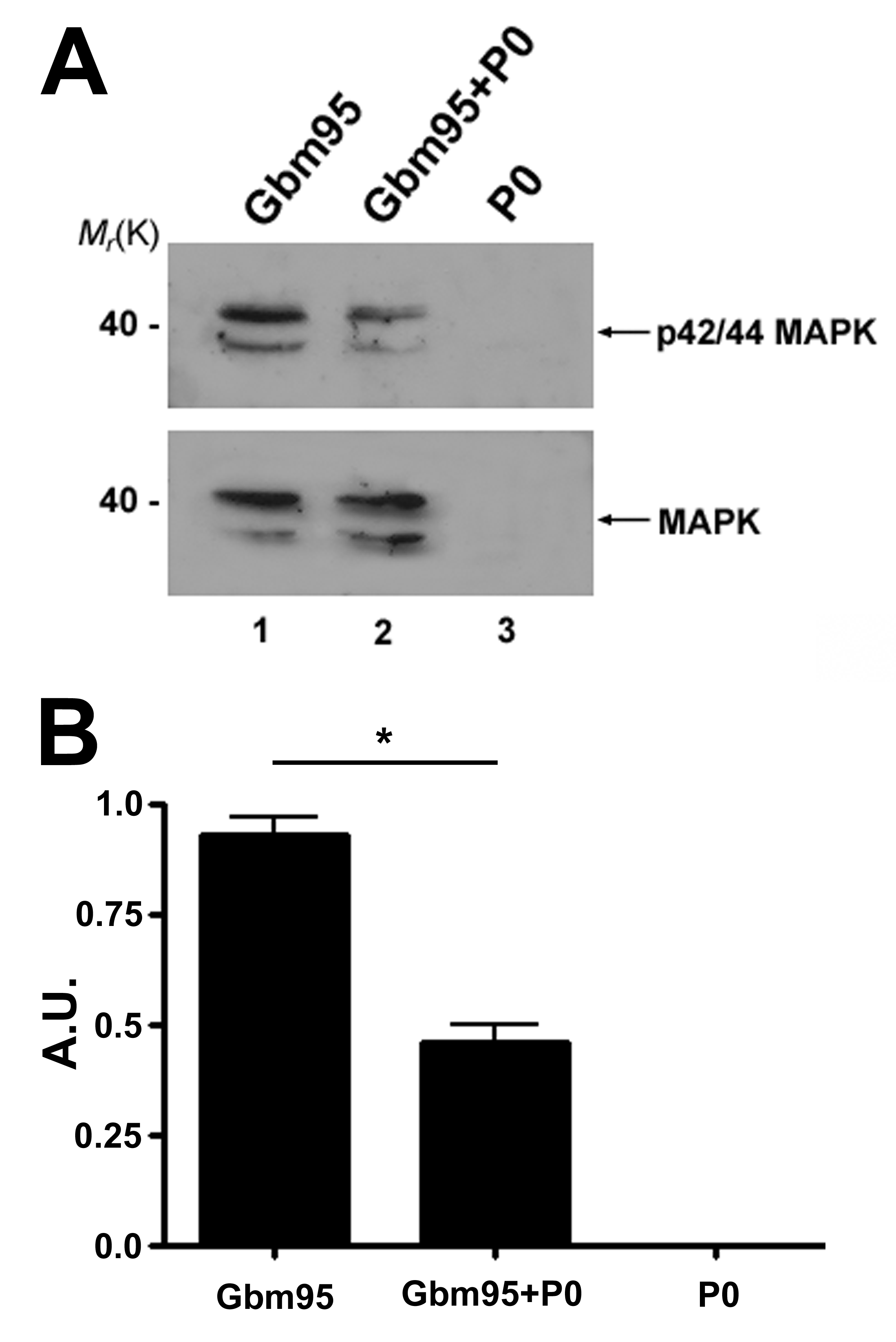

Supplement: Figure S3 — Phospho-p44/42 MAPK decreases in GBM95 cells co-cultured with P0 neurons. (A) Western blot analysis of the cell extracts from GBM95 (lane 1) and GBM95 co-cultured with P0 neurons (lane 2) and only P0 neurons (lane 3). From top to bottom, membranes were reacted with anti-phospho-p44/42 MAPK and anti-p44/42 MAPK. Histogram expressing the AU products from p44/42 MAPK over MAPK bands (B). Levels of total p44/42 MAPK did not change, but phospho p44/42 decreased in GBM95/P0 co-cultures. Molecular weight in kDa is shown on the left side of the picture. Each experiment is representative of at least three independent experiments. *p<0.05 compared to control. (TIF) [file pone.0055605.s003.tif]
